# Site-specific N-glycosylation characterization of micro monoclonal immunoglobulins based on EThcD-sceHCD-MS/MS

**DOI:** 10.3389/fimmu.2022.1013990

**Published:** 2022-09-15

**Authors:** Mengqi Luo, Yonghong Mao, Wenjuan Zeng, Shanshan Zheng, Huixian Li, Juanjuan Hu, Xinfang Xie, Yong Zhang

**Affiliations:** ^1^ Institutes for Systems Genetics, West China Hospital, Sichuan University, Chengdu, China; ^2^ Institute of Thoracic Oncology, West China Hospital, Sichuan University, Chengdu, China; ^3^ Department of Nephrology, The First Affiliated Hospital of Xi’an Jiaotong University, Xi’an, China; ^4^ Department of Laboratory Medicine, Institute of Clinical Laboratory Medicine of People’s Liberation Army (PLA), Xijing Hospital, Fourth Military Medical University, Xi’an, China

**Keywords:** N-glycosylation, monoclonal immunoglobulin, mass spectrometry, EThcD-sceHCD, micro

## Abstract

Monoclonal immunoglobulin produced by clonal plasma cells is the main cause in multiple myeloma and monoclonal gammopathy of renal significance. Because of the complicated purification method and the low stoichiometry of purified protein and glycans, site-specific N-glycosylation characterization for monoclonal immunoglobulin is still challenging. To profile the site-specific N-glycosylation of monoclonal immunoglobulins is of great interest. Therefore, in this study, we presented an integrated workflow for micro monoclonal IgA and IgG purification from patients with multiple myeloma in the HYDRASYS system, in-agarose-gel digestion, LC-MS/MS analysis without intact N-glycopeptide enrichment, and compared the identification performance of different mass spectrometry dissociation methods (EThcD-sceHCD, sceHCD, EThcD and sceHCD-pd-ETD). The results showed that EThcD-sceHCD was a better choice for site-specific N-glycosylation characterization of micro in-agarose-gel immunoglobulins (~2 μg) because it can cover more unique intact N-glycopeptides (37 and 50 intact N-glycopeptides from IgA1 and IgG2, respectively) and provide more high-quality spectra than sceHCD, EThcD and sceHCD-pd-ETD. We demonstrated the benefits of the alternative strategy in site-specific N-glycosylation characterizing micro monoclonal immunoglobulins obtained from bands separated by electrophoresis. This work could promote the development of clinical N-glycoproteomics and related immunology.

## Introduction

Human serum or plasma contains abundant disease-related glycoproteins as candidate biomarkers for diagnosis, stratification and prevention (1, 2). However, the dynamic range of serum proteins can exceed 10^9^, and most glycoproteins have very low abundance at the protein and glycan levels (1). For example, in multiple myeloma or monoclonal gammopathy of renal significance, monoclonal immunoglobulin plays an important role in the injury of target organs, but pathogenetic monoclonal immunoglobulins are complicated to purify and have low stoichiometry, despite high levels of total immunoglobulins in serum (3). Coupled with the microheterogeneity and macroheterogeneity of N-glycosylation modification, site-specific N-glycosylation (including glycoprotein, glycosite, and glycan information) analysis of these micro immunoglobulins is extremely challenging (4).

In the last few decades, researchers have made great contributions to the study of site-specific N-glycosylation of the human serum glycoproteome. For example, immunodepletion technologies and ProteoMiner protein enrichment methods have been used to remove abundant plasma proteins (5–7). Many enrichment materials have been developed to remove nonglycopeptides in plasma (4, 8). These methods and materials are beneficial to the study of serum glycoproteomics. For targeted serum glycoproteins, there are still many difficulties in glycoprotein preparation, mass spectrometry analysis, data processing, etc (9–11). The classical research methods are as follows: First, tens to hundreds of micrograms of serum proteins were purified and digested into peptides. Tens to hundreds of micrograms of proteins were needed as a starting amount because only 2~5% of the total peptide contents were glycopeptides. Second, intact N-glycopeptides were enriched using hydrophilic interaction liquid chromatography (HILIC) or other enrichment materials to avoid interference from non-glycopeptides. Third, enriched intact N-glycopeptides should be analyzed by advanced LC-MS/MS and professional software (12). However, the content of many disease-related serum proteins is difficult to reach tens to hundreds of micrograms. To solve this problem, recombinant expression of the target glycoprotein in human derived cells can provide sufficient protein. Nonetheless, it is time consuming and laborious, and may be difficult to detect disease-related glycosylation changes (13). The other strategy is to isolate and purify one target glycoprotein from enough serum. For example, Yu et al. used jacalin affinity chromatography, a protein G affinity column, and Sephacryl S-300 gel filtration chromatography to obtain purified polymer IgA1 and monomer IgA1. However, they need to consume hundreds of milliliters of blood per patient (14). In addition, the glycoprotein based on an antibody affinity purification strategy also has difficult meeting the common requirement for protein content unless industrialized (15). For micro proteins, only a few glycosylation modifications can be analyzed. Hence, we urgently need a strategy for in-depth and site-specific N-glycosylation analysis of micro proteins.

In recent years, a novel fragmentation mode (EThcD-sceHCD) has appeared as a valuable approach for glycoproteomics (16). EThcD means fragment parent ions *via* ETD first, and then the products ions were fragmented *via* HCD (17). sceHCD means the stepped collision energy HCD (10). EThcD-sceHCD means an alternative fragmentation which switch modes between the EThcD and sceHCD in a duty cycle. This combined fragmentation strategy capitalized on the advantages of both EThcD and sceHCD to produce two types of MS2 spectra (EThcD spectra and HCD spectra). In other words, EThcD-sceHCD can produce more rich and useful ions for the accurate identification of intact glycopeptides than EThcD and sceHCD alone. For example, site-specific N/O-glycosylation of the HIV-1 Env protein gp120 was characterized by EThcD-sceHCD because EThcD-sceHCD can increase the number of identified glycopeptides and produce more comprehensive fragment ions. Moreover, EThcD-sceHCD had a higher Byonic score in the top-scoring identification than did the sceHCD and EThcD spectra of the same intact N-glycopeptide (16). Site-specific N-glycosylation patterns of serum IgGs based on EThcD-sceHCD revealed that intact N-glycopeptides were differentially expressed in healthy controls (HCs) and chronic kidney disease (CKD) patients (18). In addition, EThcD-sceHCD can be used for complex clinical sample (e.g., plasma, urine, cells, and tissues) glycosylation studies and outperforms previous approaches (EThcD, sceHCD, HCD-pd-ETD, and sceHCD-pd-ETD) in the balance of depth and accuracy of intact N-glycopeptide identification (19, 20). However, these comparative studies used tens to hundreds of micrograms of proteins based on filter-aided sample preparation (FASP) digestion and intact glycopeptide enrichment strategies. We do not know if EThcD-sceHCD is more applicable to micro in-gel proteins than other methods.

Herein, we aim to provide an alternative strategy for site-specific N-glycosylation analysis of micro disease-related monoclonal immunoglobulins (1~10 µg) based on in-gel digestion and without intact glycopeptide enrichment. More precisely, we integrated human serum monoclonal IgA and IgG preparations based on the HYDRASYS system, and LC-MS/MS analysis after in-agarose-gel digestion and compared the intact N-glycopeptide identification performance of different mass spectrometry dissociation methods (EThcD-sceHCD, sceHCD, EThcD and sceHCD-pd-ETD). Finally, we determined that EThcD-sceHCD is a better choice for site-specific N-glycosylation characterization of micro in-gel immunoglobulins.

## Experimental section

### Biospecimen collection

This study was approved by the medical ethics committee at the First Affiliated Hospital of Xi’an Jiaotong University, and conducted in adherence to the Declaration of Helsinki. Serum samples from patients with multiple myeloma were collected and stored at -80°C before use. Written informed consent was collected.

### Preparation of microscale glycoproteins

Serum samples from two patients with multiple myeloma were collected. Serum monoclonal IgA and IgG were purified by using the HYDRAGEL 4 IF kit (Sebia, Cedex, France) in the semiautomated agarose electrophoresis and immunofixation HYDRASYS system. We modified the manufacturer’s instructions for monoclonal immunoglobulin purification in the HYDRASYS system. In brief, for protein electrophoresis, a 10 μL volume of 2-4 times diluted serum samples was applied manually to the applicator wells. The applicator was placed into the wet storage chamber with the teeth up and samples were allowed to diffuse into teeth for 5 min. Then protein migration was completed in the HYDRASYS system with the applicators placed. After electrophoresis, all wells were incubated with fixative solution, except one with IgA antiserum or IgG antiserum as positive control. Next, we removed the remaining fixative solution and antiserum by filter paper, washed the agarose gel with HYDRASYS wash solution three times and stained it with amidoblack stain for 15 minutes at room temperature. Distaining was performed using 0.1% acetic acid and ddH_2_O. Finally, referring to the control band with IgA or IgG antiserum incubation, the bands of monoclonal IgA or IgG (~2 μg) were excised from lanes fixed only with fixative solution.

### Decolorization, reduction, alkylation and digestion

The monoclonal IgA and IgG glycoproteins (~2 μg) were proteolyzed using an in-agarose-gel digestion protocol. Briefly, the bands of monoclonal IgA or IgG were cut into 1 mm^3^ pieces. A total of 150 μL of destainer (50% ACN in 50 mM NH_4_CO_3_ buffer) was used for shock decoloration. Then, 150 μL of 100% ACN was added for dehydration. After carrying out the reduction reaction by adding 100 μL of 20 mM DTT for 45 min at 56°C, the alkylation reaction was carrying out by adding 100 μL of 50 mM IAA and incubating in the dark for 30 min. Then, 200 μL of destainer and 100% ACN were used to wash out these salts. These microscale glycoproteins were digested for 18 h at 37°C after adding 50 μL of trypsin (10 ng/μL) to each filter tube. The peptides were collected and combined by washing three times with 200 μl of buffer A (1% FA, 2% ACN), buffer B (1% FA, 50% ACN), and buffer C (100% ACN). Meanwhile, all samples were processed by ultrasound (Kunshan, China) every time. The ultrasound instrument was set as follows: power = 100 W; frequency = 25 KHZ; total time = 10 min; on = 7 s; off = 3 s. The processed samples were centrifuged at 13,000×g for 15 min at 4°C. The peptides were dried and stored at -80°C.

### LC-MS/MS analysis

The peptides from each microscale glycoprotein were resuspended in 20 μL of 0.1% FA individually. Four microliters of peptides were separated on a column (ReproSil-Pur C18-AQ, 1.9 μm, 75 μm inner diameter, length 20 cm; Dr Maisch) over a 30-min gradient (0–2 min, 5–12% B; 2–7 min, 12–22% B; 7–21 min, 22–32% B; 21–22 min, 32–90% B; 22–30 min, 90% B) at a flow rate of 350 nL/min, and analyzed on an Orbitrap Fusion Lumos Mass Spectrometer (Thermo Fisher, USA). Four different fragmentation modes (EThcD-sceHCD, sceHCD, EThcD and sceHCD-pd-ETD) were used for peptide analysis. The parameter details of these modes can be found in our previous descriptions (19).

Specifically, EThcD-sceHCD-MS/MS was performed using an alternative fragmentation between the EThcD and sceHCD modes in a duty cycle (3 s). In the EThcD duty cycle (2 s), MS1 was analyzed using 800–2000 m/z at an Orbitrap resolution of 60,000. The RF lens, AGC target, maximum injection time (MIT), and exclusion duration were 40%, 2.0 e5, 50 ms, and 15 s, respectively. MS2 was analyzed with 2 m/z at an Orbitrap resolution of 30,000. The AGC target, MIT, and EThcD type were standard, 150 ms, and 35%, respectively. In the sceHCD duty cycle (1 s), MS1 was analyzed using 800–2000 m/z at an Orbitrap resolution of 60,000. The RF lens, AGC target, MIT and exclusion duration were 40%, standard, auto, and 15 s respectively. MS2 was analyzed with 1.6 m/z at an Orbitrap resolution of 30,000. The AGC target, MIT, and HCD collision energies were 200%, auto, and 30%, respectively. In addition, the sceHCD mode was turned on with an energy difference of ±10% (20-30-40%).

### Data analysis

The RAW data files were searched using Byonic software (version 3.10.10, Protein Metrics, Inc.). The human IgG or IgA Uniprot database was chosen. The mass tolerances for precursors and fragment ions were set as ± 6 ppm and ± 20 ppm, respectively. The fragmentation type was set as “Both HCD & EThcD”, “HCD”, “EThcD” or “Both HCD & ETD”. Two missed cleavage sites were allowed. Carbamidomethyl (C) was set as fixed modification. Oxidation (M) and Acetyl (Protein N-term) were set as variable modifications. The “182 human N-glycans” was set as the N-glycan modification. Protein groups were filtered to 1% FDR. Quality control methods for intact N-glycopeptide identification included a Byonic score of over 200, a logProb value of over 2, and at least 5 amino acids. Each spectrum of intact N-glycopeptide should be confirmed manually by checking the oxonium ions and b/y/c/z ions to ensure the correct identification of the peptide sequence and attached glycan compositions. The RAW Data can be obtained *via* ProteomeXchange with identifier PXD035757.

## Results and discussion

Multiple myeloma (MM), as the second most common adult hematological malignancy with skeletal components as its primary site, is a cancer characterized by the proliferation of monoclonal plasma cells derived from the bone marrow (21). Recently, N-glycosylation of immunoglobulin G or A was shown to have a significant correlation with the development of many cancer types (22–24). Several published studies have investigated the N-glycosylation profiles of serum total or polyclonal IgA or IgG in MM (25). However, the N-glycosylation profiles and their potential prognostic value, as well as the pathogenesis of specific N-glycosylation of monoclonal immunoglobulin involved in MM remain unclear. Therefore, more research is needed to modify the purification method of monoclonal immunoglobulin and explore their site-specific N-glycosylation analysis by mass spectrometry.

**Figure 1:**
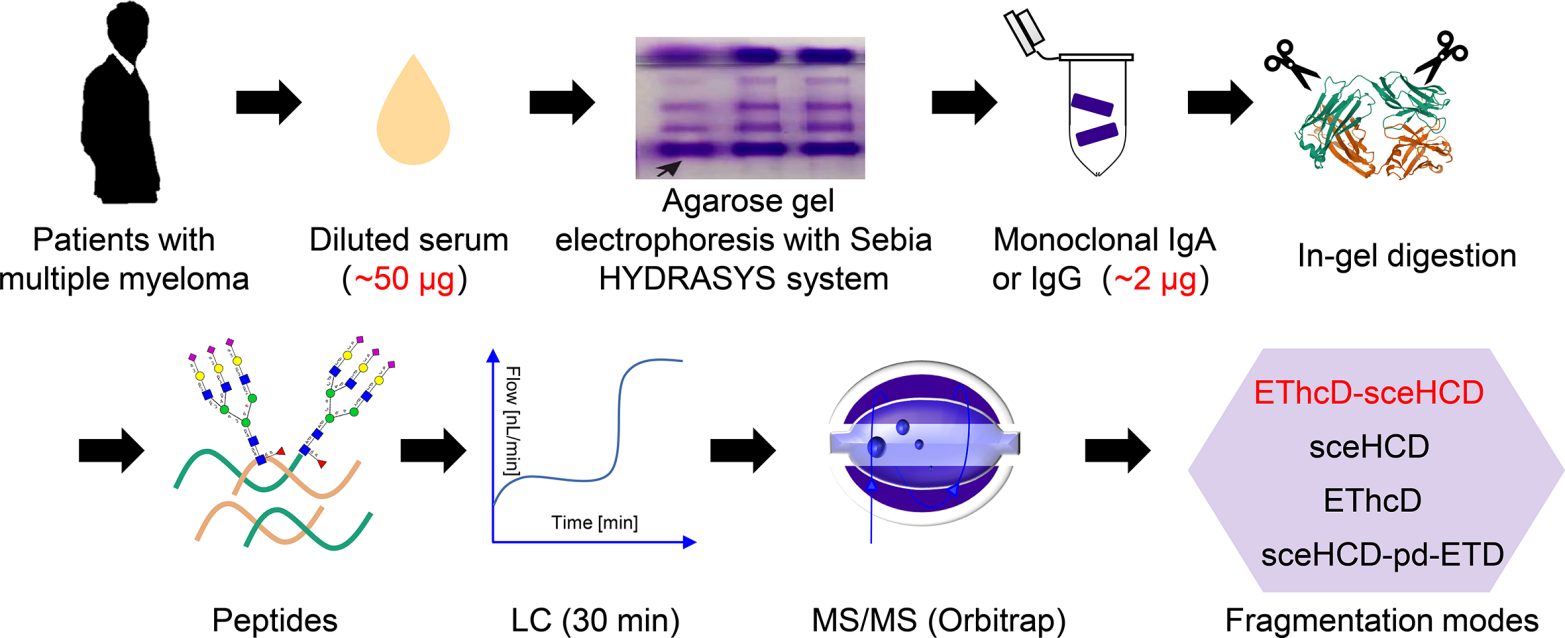
Schematic representation of the workflow for the intact N-glycopeptide analysis of microscale human serum monoclonal IgA and IgG from patients with multiple myeloma using four different dissociation methods.

Over the past year, we have proven that EThcD-sceHCD has a better performance than other dissociation methods in the intact glycopeptide analysis of HIV-1 gp120, IgG subclasses, and complex clinical samples (16, 18–20). However, it remains to be seen whether EThcD-sceHCD is more applicable to micro in-gel immunoglobulins from human serum than other methods. For this purpose, the following experiment was designed (**Figure 1**). We collected serum samples from MM patients and purified monoclonal IgG or IgA in the HYDRASYS system. The baseline characteristics of two patients with multiple myeloma included in the study were shown in **Table 1**. Monoclonal IgG or IgA from MM patients will appear as a clear band on the agarose gel, unlike normal human serum polyclonal immunoglobulin protein with a diffuse band. After reducing, alkylating, and digesting the target band, peptides were obtained and analyzed by LC-MS/MS with a 30-min separation gradient and four different dissociation methods (EThcD-sceHCD, sceHCD, EThcD and sceHCD-pd-ETD).

**Table 1:** Baseline characteristics of patients with multiple myeloma included in the study.

Characteristics	Patient 1	Patient 2
**Age (ys)**	72	63
**Gender**	Male	Male
**Monoclonal immunoglobulin type**	IgA kappa	IgG lambda
**Concentration of monoclonal immunoglobulin (g/l)**	43.0165	94.64
**IgG (g/L)**	3.05	98.7
**IgA (g/L)**	68.2	<0.27
**IgM (g/L)**	<0.19	<0.19
**C3 (g/L)**	0.5	0.32
**C4 (g/L)**	0.08	<0.07
**Lambda chain (g/L)**	0.36	25.5
**Kappa chain (g/L)**	3.24	0.18
**Free lambda chain (mg/L)**	8.44	46.9
**Free kappa chain (mg/L)**	21.9	5.25
**Free Lambda/Kappa**	2.59	0.11
**Serum albumin (g/L)**	28.7	15.5
**Serum creatinine (mmol/L)**	68	68
**Hemoglobinuria (g/L)**	100	69
**Serum β2 microglobulin**	4560	3271.6
Abnormal plasma cell (%)*	27	23
**ISS stage#**	II	II

*Abnormal plasma cell by bone marrow biopsy; # The International Staging System (ISS).

Monoclonal rather than polyclonal IgG and IgA were chosen because monoclonal immunoglobulins are more associated with the development of MM. To purify monoclonal IgG and IgA, the HYDRASYS system was used rather than SDS-PAGE. The reason is that SDS-PAGE cannot distinguish monoclonal and polyclonal IgG or IgA. However, the HYDRASYS system can display unique monoclonal immunoglobulin band scanning features with the advantages of high resolution, good precision, simple and fast. It is worth noting that the content of monoclonal IgG or IgA in an agarose band is very low (~2 μg). The content is well below the amount of protein previously required for glycoproteomics (26). Hence, we presented an integrated workflow for micro serum monoclonal IgA and IgG preparation in the HYDRASYS system, in-agarose-gel digestion, LC-MS/MS analysis without intact N-glycopeptide enrichment, and compared the identification performance of different mass spectrometry dissociation methods (EThcD-sceHCD, sceHCD, EThcD and sceHCD-pd-ETD) (**Figure 1**). N-glycosylation is a very complex post-translational modification. Intact N-glycopeptide characterization based on advanced LC-MS/MS can probe microheterogeneity by localizing N-glycans to asparagine residues (N-X-S/T/C, X≠P). Hence, it is becoming a major part of modern N-glycoproteomic analysis (10).

In this study, we selected serum samples from MM patients diagnosed with monoclonal IgA lambda and IgG kappa by immunofixation electrophoresis. Theoretically, each monoclonal immunoglobulin should have just one specific heavy chain and one matched light chain. Our EThcD-sceHCD mass spectrometry results using the micro proteins showed that both monoclonal immunoglobulins had high abundance and coverage (IgA1 with 98.9% relative abundance and 82.72% coverage, IgG2 with 99.6% relative abundance and 87.12% coverage) in the total detected proteins, which supported the high degree of purity in the agarose gel band with our modified purification method by using the HYDRASYS system (**Supplementary Materials**). Previous studies have shown that IgA1 contains two N-glycosites (N144 and N340), and IgG2 contains one N-glycosite (N174) (27). In this study, we specifically identified and compared the data of all N-glycosites in equal quantities of micro monoclonal IgA1 and IgG2 by EThcD-sceHCD, sceHCD, EThcD and sceHCD-pd-ETD. However, the N-glycosite (N144) in IgA1 can only be identified by EThcD-sceHCD and sceHCD. EThcD and sceHCD-pd-ETD missed this N-glycosite probably because it is too poorly glycosylated in MM patients (**Supplementary Materials**). Furthermore, we compared the N-glycosylation modifications of human serum monoclonal IgA1 and IgG2 using four different dissociation methods. As shown in **Table 1**, 37 unique intact N-glycopeptides of IgA1 with 124 N-glycoPSMs and 24 N-glycans were identified by EThcD-sceHCD. Meanwhile, there were 50 unique IgG2 intact N-glycopeptides with 119 N-glycoPSMs and 27 N-glycans were identified by EThcD-sceHCD. The order in terms of the number of identifications was EThcD-sceHCD, sceHCD, sceHCD-pd-ETD and EThcD. Hence, the results indicated that EThcD-sceHCD outperformed the other three common modes (EThcD, sceHCD and sceHCD-pd-ETD) in the depth of N-glycoPSM, N-glycan, and intact N-glycopeptide identification with micro protein (**Table 2**).

**Table 2:** Comparison of the N-glycosylation modifications of human serum monoclonal IgA1 and IgG2 from patients with multiple myeloma using four different dissociation methods.

Dissociation mode	EThcD-sceHCD		sceHCD		EThcD		sceHCD-ETD	
Sample type	IgA1	IgG2	IgA1	IgG2	IgA1	IgG2	IgA1	IgG2
Number of N-glycoPSMs	**124**	**119**	73	70	21	15	27	30
Number of N-glycans	**24**	**27**	23	27	7	13	14	17
Number of intact N-glycopeptides	**37**	**50**	32	44	9	15	16	26

The bold values means the number of identifiers under EThcD-sceHCD fragmentation mode is highlighted.

To our knowledge, sceHCD has been the most popular mode for intact N-glycopeptides because it generally provides higher quality spectra and scanning speed. Special software, such as pGlyco2.0, has been developed to analyze the data produced (28). In addition, EThcD can be more suitable for site-specific O-glycosylation and intact N-glycopeptides with more than one glycosite analyses because it can retain intact glycan moieties with few glycan dissociation events (29, 30). However, sceHCD-pd-ETD may be suitable for intact N/O-glycopeptides from complex samples without enrichment analyses because it makes use of glycopeptide-specific oxonium ions derived from glycan fragmentation scans to trigger subsequent ETD fragmentation (31). Until recently, EThcD-sceHCD has arguably become the most dazzling N/O-glycoproteomic method because it makes full use of the advantages of both sceHCD and EThcD (16). Compared with EThcD, sceHCD, HCD-pd-ETD, and sceHCD-pd-ETD, EThcD-sceHCD has been determined to be applicable and superior in site-specific glycosylation analyses of simple glycoproteins (IgGs and gp120) and complex clinical samples (plasma from prostate cancer patients, urine from immunoglobulin A nephropathy patients, human hepatocarcinoma cell lines, and thyroid tissues from thyroid cancer patients) (16, 18, 20). Therefore, we are the first to explore whether EThcD-sceHCD is more suitable for the site-specific N-glycosylation analysis of micro in-agarose-gel proteins in this study.

**Figure 2:**
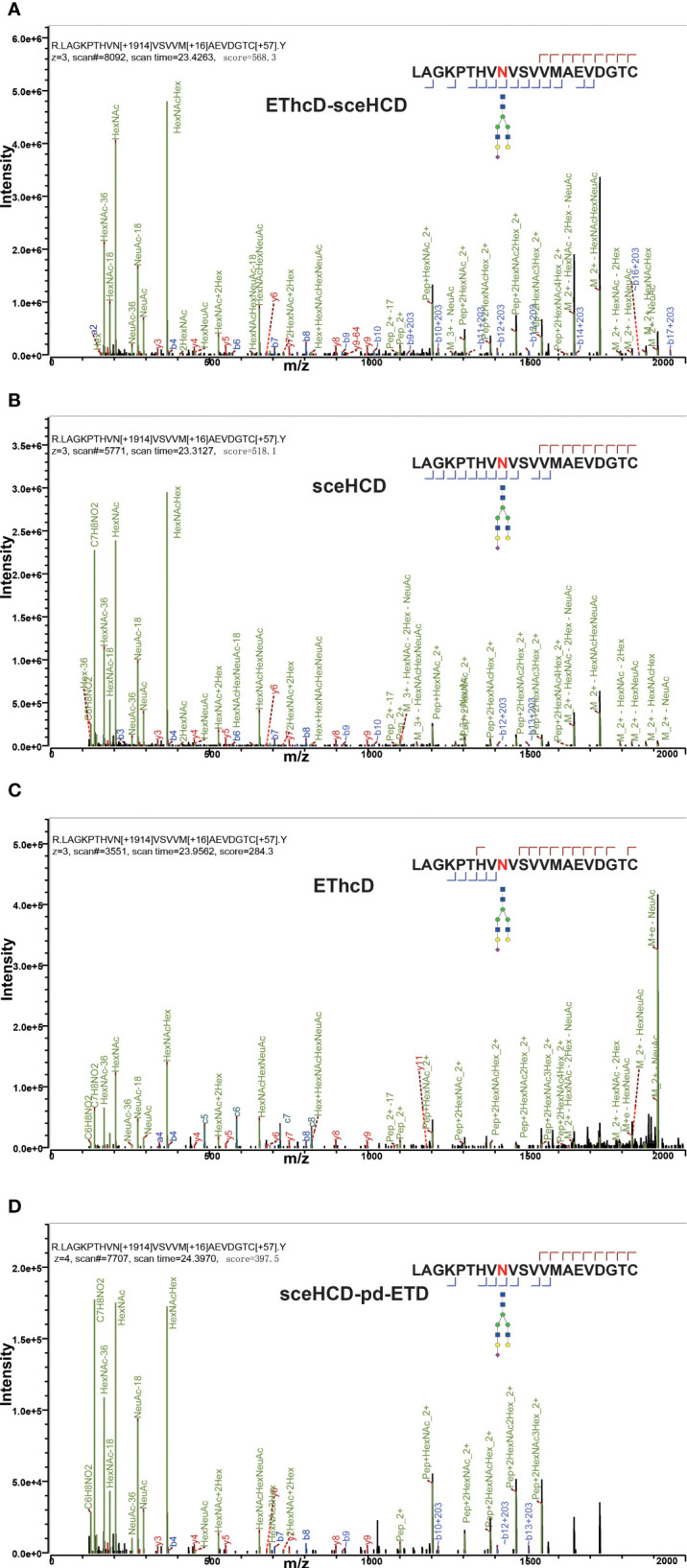
Comparison of EThcD-sceHCD **(A)**, sceHCD **(B)**, EThcD **(C)**, and sceHCD-pd-ETD **(D)** spectra of one intact N-glycopeptide (^322^LAGKPTHV**N**VSVVMAEVDGTC^352^-HexNAc(4)Hex(5)NeuAc(1)) from serum monoclonal IgA1.

**Figure 3:**
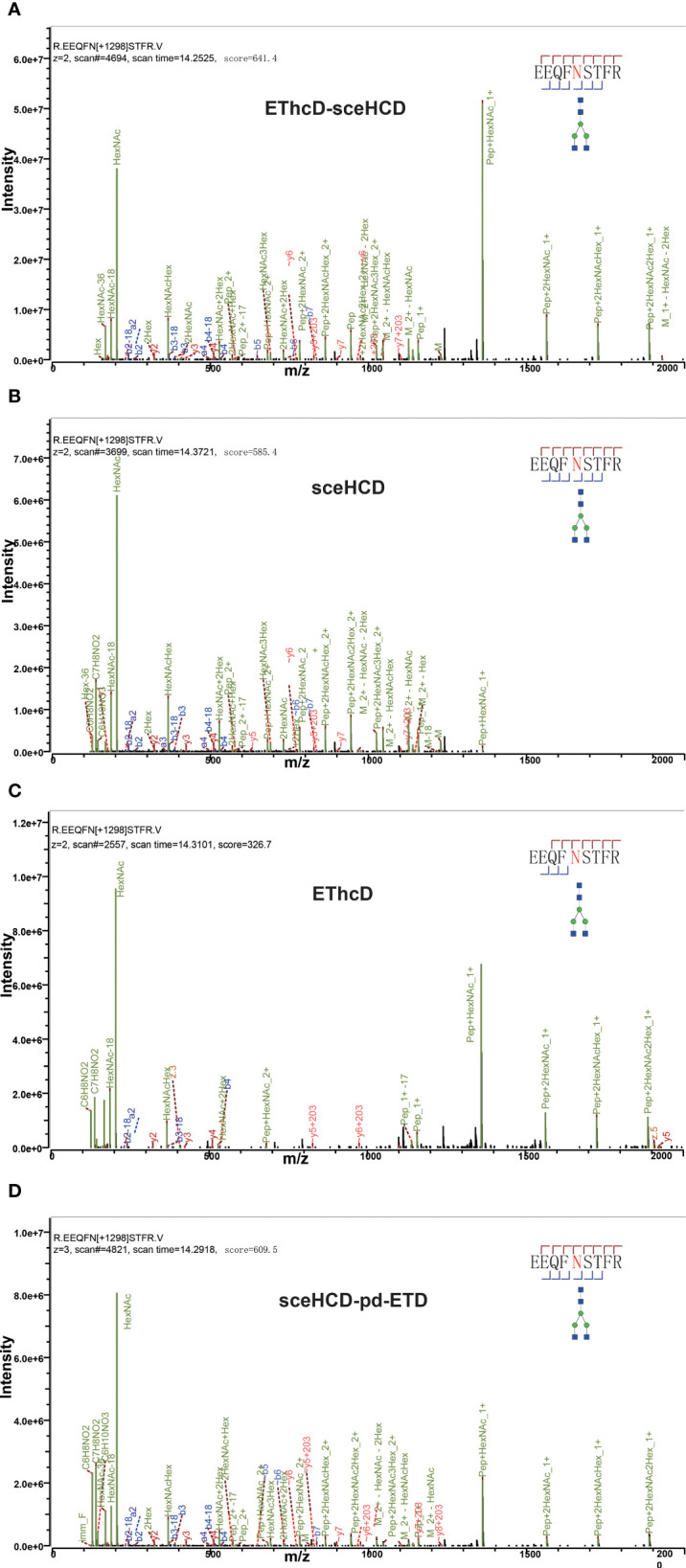
Comparison of EThcD-sceHCD **(A)**, sceHCD **(B)**, EThcD **(C)**, and sceHCD-pd-ETD **(D)** spectra of one intact N-glycopeptide [^172^EEQF**N**STFR^180^-HexNAc(4)Hex(3)] from serum monoclonal IgG2.

Different fragmentation methods can produce abundant fragment ions with distinct features. For example, all methods provided confident N-glycosite (N340) localization and abundant information about the N-glycan composition [HexNAc(4)Hex(5)NeuAc(1)] of a peptide (^322^LAGKPTHV**N**VSVVMAEVDGTC^352^) from human serum monoclonal IgA1 (**Figure 2**). Their spectra included glycan fragments, b/y or/and c/z-type peptide backbone fragments, Y ions, and so on. Hence, all of these methods can correctly analyze this intact N-glycopeptide. However, when we compared these spectra in detail, EThcD-sceHCD provided more ion information with a larger Byonic Score (568.3). As the “raw” indicator of PSM correctness, it reflects the absolute quality of the peptide-spectrum match (**Figure 2**). The same holds true for IgG2. All methods provided confident N-glycosite (N174) localization and abundant information about the N-glycan composition [HexNAc(4)Hex(3)] of a peptide (^172^EEQF**N**STFR^180^) from human serum monoclonal IgG2 (**Figure 3**). The spectrum of EThcD-sceHCD also had a larger Byonic Score (641.4). In addition, only EThcD-sceHCD and sceHCD can provide confident N-glycosite (N144) localization in IgA1. Considering the number of N-glycoPSMs, N-glycans, and intact N-glycopeptides identified by these methods, we canconclude that EThcD-sceHCD is a better choice for site-specific N-glycosylation analysis of micro in-gel proteins.

**Figure 4:**
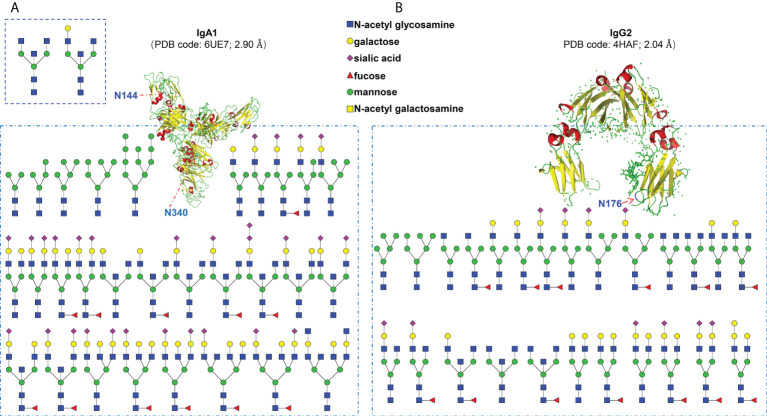
Site-specific N-glycosylation profiling of human serum monoclonal IgA1 **(A)** and IgG2 **(B)** from patients with multiple myeloma.

Finally, we profiled the site-specific N-glycosylation of serum monoclonal IgA1 and IgG2 from patients with MM based on the comprehensive glycoproteomic information obtained in this work. As shown in **Figure 4A**, the N144 site of IgA1 was modified by 2 kinds of N-glycans. The other N340 site of IgA1 was modified by 29 kinds of N-glycans. For IgG2, there were 25 different N-glycan compositions attached to the N174 site (**Figure 4B**). These results imply that our strategy can decipher the microheterogeneity and macroheterogeneity of N-glycosylation from micro in-gel proteins.

## Conclusions

Site-specific N-glycosylation of micro proteins has always been a challenge but an important research hotspot in the glycoproteomics field. Herein, we proved the reliability and superiority of EThcD-sceHCD in the site-specific N-glycosylation characterization of micro in-agarose-gel immunoglobulins (~2 μg). By integrating serum monoclonal IgA and IgG from patients with multiple myeloma preparation in the HYDRASYS system and in-agarose-gel digestion without intact N-glycopeptide enrichment, we compared the identification performance of four different mass spectrometry dissociation methods (EThcD-sceHCD, sceHCD, EThcD and sceHCD-pd-ETD). We proved that EThcD-sceHCD performed better in the identification number of N-glycoPSMs, N-glycans and intact N-glycopeptides. In addition, it can provide more high-quality spectra than sceHCD, EThcD and sceHCD-pd-ETD. Hence, we presented an alternative strategy in site-specific N-glycosylation characterizing micro disease-related immunoglobulins obtained from bands separated by electrophoresis. This study may provide a robust method of N-glycosylation analysis for micro glycoproteins and promote the development of research on the N-glycoproteome.

## Data availability statement

The datasets presented in this study can be found in online repositories. The names of the repository/repositories and accession number(s) can be found below: ProteomeXchange *via* accession ID: PXD035757.

## Ethics statement

This study was approved by the medical ethics committee at the First Affiliated Hospital of Xi’an Jiaotong University, and conducted in adherence to the Declaration of Helsinki. The patients/participants provided their written informed consent to participate in this study.

## Author contributions

YZ and XX designed the research; ML, YM, SZ, WZ, HL, and JH processed the samples and performed LC-MS/MS analysis; YZ and XX performed data analysis and wrote the manuscript. All authors have approved the final version of the manuscript.

## Funding

We are grateful for financial support from the National Natural Science Foundation of China (31901038, 81800639), and the Department of Science and Technology of Sichuan Province (2021YJ0479).

## Conflict of interest

The authors declare that the research was conducted in the absence of any commercial or financial relationships that could be construed as a potential conflict of interest.

## Publisher’s note

All claims expressed in this article are solely those of the authors and do not necessarily represent those of their affiliated organizations, or those of the publisher, the editors and the reviewers. Any product that may be evaluated in this article, or claim that may be made by its manufacturer, is not guaranteed or endorsed by the publisher.
